# Neuron-specific regulation of superoxide dismutase amid pathogen-induced gut dysbiosis

**DOI:** 10.1016/j.redox.2018.05.007

**Published:** 2018-05-19

**Authors:** Alexander M. Horspool, Howard C. Chang

**Affiliations:** Department of Biological Sciences, Binghamton University, SUNY, Binghamton, NY 13902, USA

## Abstract

Superoxide dismutase, an enzyme that converts superoxide into less-toxic hydrogen peroxide and oxygen, has been shown to mediate behavioral response to pathogens. However, it remains largely unknown how superoxide dismutase is regulated in the nervous system amid pathogen-induced gut dysbiosis. Although there are five superoxide dismutases in *C. elegans*, our genetic analyses suggest that SOD-1 is the primary superoxide dismutase to mediate the pathogen avoidance response. When *C. elegans* are fed a *P. aeruginosa* diet, the lack of SOD-1 contributes to enhanced lethality. We found that guanylyl cyclases GCY-5 and GCY-22 and neuropeptide receptor NPR-1 act antagonistically to regulate SOD-1 expression in the gustatory neuron ASER. After *C. elegans* ingests a diet that contributes to high levels of oxidative stress, the temporal regulation of SOD-1 and the SOD-1–dependent response in the gustatory system demonstrates a sophisticated mechanism to fine-tune behavioral plasticity. Our results may provide the initial glimpse of a strategy by which a multicellular organism copes with oxidative stress amid gut dysbiosis.

## Graphical abstract

**Figure:**
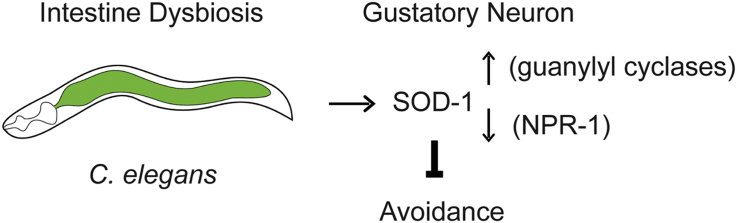

## 1 Introduction

Gut dysbiosis is caused by an imbalance of beneficial and harmful microbes in the intestine [1], [2]. In addition to digestive tract–related morbidity [2], [3], [4], [5], [6], recent studies have revealed that dysbiosis of gut microbiota plays a role in emotional and cognitive behaviors [7], [8], [9], [10] and is found in patients with neurodegenerative diseases [11], [12], [13].

In the laboratory, *C. elegans* is often reared on a petri dish that contains a lawn of non-pathogenic *Escherichia coli* OP50 [14]. Recent studies reveal that pathogenic bacteria *Pseudomonas aeruginosa*, which are frequently found in the habitats of nematodes [15], [16], can colonize the intestine of *C. elegans* [17], [18], [19], [20]. Switching *C. elegans* from a diet of *E. coli* to a diet of *P. aeruginosa* creates a condition that mimics gut dysbiosis [21], [22], [23]. The change in the microbial composition in the intestine activates host responses [22], [24], [25], [26]. For example, intestinal accumulation of *P. aeruginosa* activates the production of reactive oxygen species (ROS) [22], [27], [28], [29]. The ROS burst further elevates the expression of antioxidant enzymes, including superoxide dismutase SOD-1 [22]. SOD-1 is an enzyme that converts superoxide into less-toxic hydrogen peroxide and oxygen [30], [31], [32]. Indeed, animals carrying a *sod-1* deletion elicit a strong aversive response to *P. aeruginosa*, presumably due to their reduced capacity to ameliorate elevated ROS [22].

ASE neurons are a pair of chemosensory neurons defined by their ability to detect water-soluble cues and transmit this information to evoke a specific behavioral response [33]. Therefore, they are often regarded as the gustatory (taste) neurons in *C. elegans*. Although ASE neuron cell bodies are symmetrically positioned in the lateral ganglia region of *C. elegans* brain, the molecular compositions of left (ASEL) and right (ASER) gustatory neurons are not identical [34], [35]. ASEL and ASER gustatory neurons express distinct members of a putative chemoreceptor gene family and respond in distinct manners to different cues [36], [37]. For instance, animals devoid of the ASER neuron elicited a heightened aversive response to *P. aeruginosa* [22]. In contrast, animals lacking the ASEL neuron did not show the heightened pathogen avoidance response. SOD-1 is present in the nervous system, and SOD-1 expression is elevated in the ASER neuron by *P. aeruginosa*. It is possible that ASER-specific chemosensory receptors activate SOD-1, but experimental data have yet to support this notion.

Another poorly understood phenomenon concerns the following observation: After extended feeding on *P. aeruginosa*, ASER-specific SOD-1 elevation becomes diminished. Reduction of SOD-1 is coupled with the initiation of aversive behavior to *P. aeruginosa* [22]. However, the mechanism of SOD-1 down-regulation after extended *P. aeruginosa* feeding remains unknown. It has been shown that deletion in neuropeptide receptor NPR-1 results in extended feeding on *P. aeruginosa* [21], [26]. Neuropeptide receptor NPR-1 is *C. elegans* neuropeptide Y receptor, which regulates food satiety and stress response [21], [26], [38], [39]. The NPR-1 receptor is present in the ASER neuron [40]. We hypothesize that NPR-1 may play a role in modulating the SOD-1–dependent behavioral response to *P. aeruginosa* by down-regulating SOD-1 expression in the ASER neuron.

There are three zinc–copper superoxide dismutase isoforms (SOD-1, SOD-4, and SOD-5) and two manganese superoxide dismutase isoforms (SOD-2 and SOD-3) in *C. elegans* [41]. Based on their subcellular localization, the zinc–copper isoforms are further classified as cytoplasmic (i.e., SOD-1 and SOD-5) and extracellular/secreted (i.e., SOD-4) superoxide dismutases. Previous studies suggest these isoforms are expressed rather ubiquitously, including in the nervous system. For example, SOD-1 appears to be expressed in most cells of the worm [42], [43]. SOD-5 expression is inducible and has been detected in a small subset of neurons [42]. SOD-1 has been previously suggested to act in the gustatory neuron ASER to regulate *C. elegans* behavioral response to *P. aeruginosa* [22]. Do additional superoxide dismutases in *C. elegans* mediate the behavioral response to *P. aeruginosa*? And what are the molecular mechanisms that regulate the superoxide dismutase–dependent response to pathogen-induced gut dysbiosis?

In this study, we found that SOD-1 and SOD-5 are both present in the gustatory neuron ASER. Our genetic analyses suggest that SOD-1 plays a primary role in regulating the behavioral response to *P. aeruginosa*. SOD-1 is induced in the nervous system and in the intestine by oxidative stress. Lack of SOD-1 contributes to enhanced lethality after *C. elegans* is fed the *P. aeruginosa* diet. ASER-specific guanylyl cyclases GCY-5 and GCY-22 activate SOD-1 expression, whereas neuropeptide receptor NPR-1 promotes the reduction of SOD-1 in the ASER neuron. Therefore, we have identified the regulatory mechanisms that contribute to the activation and reduction of superoxide dismutase. Our results also suggest that zinc–copper superoxide dismutase plays a major role in mediating the behavioral response amid pathogen-induced gut dysbiosis.

## 2 Results

### 2.1 Superoxide dismutase isoform SOD-1 plays a primary role in behavioral response to *P. aeruginosa*

**Fig. 1:**
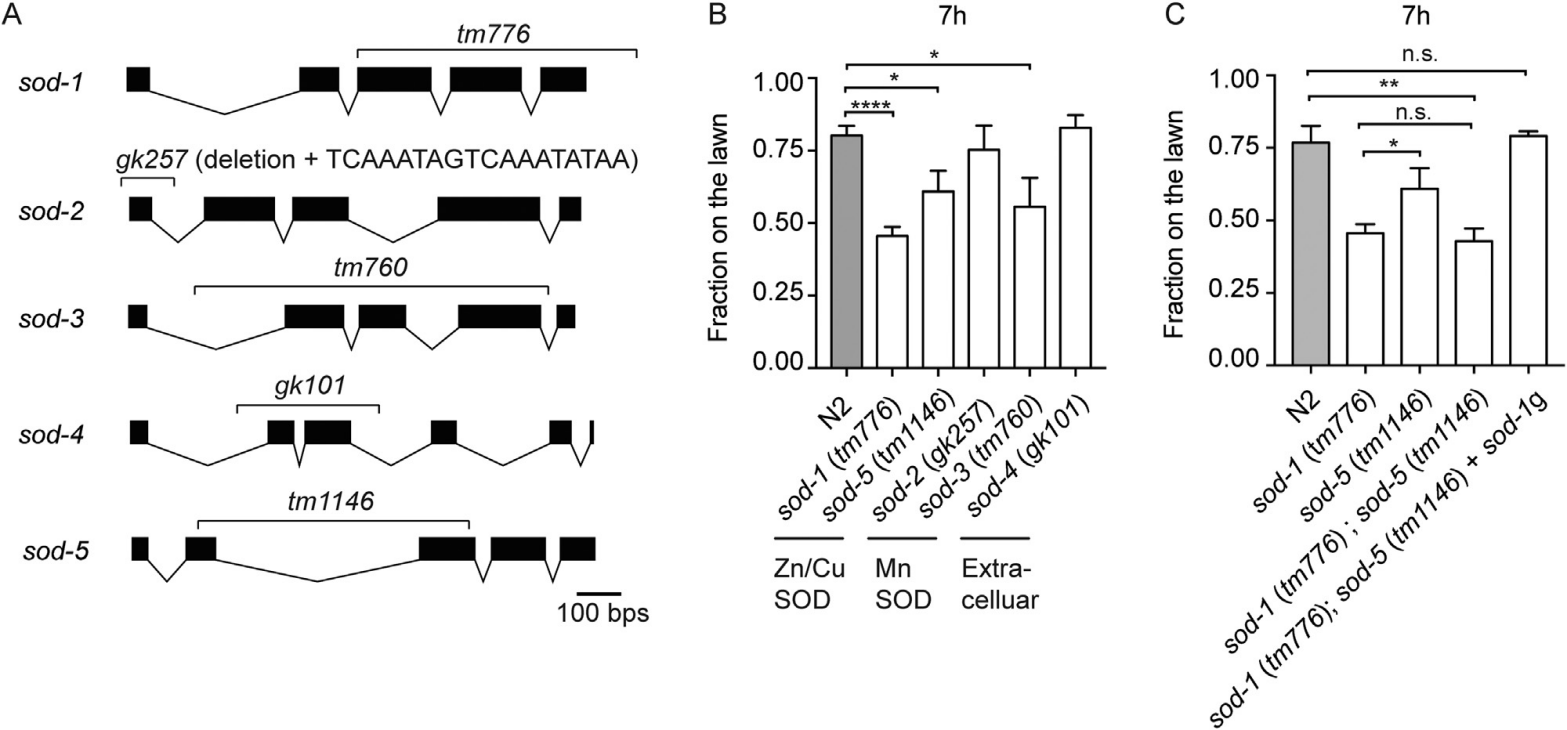
Deletions in superoxide dismutase isoforms elicit heightened avoidance response to *P. aeruginosa* PA14. (A) The deleted regions of the *sod-1*, *sod-2*, *sod-3*, *sod-4*, and *sod-5* mutations used in this study are indicated. Black box indicates an exon. Connected lines indicate the region of an intron. (B) After 7 h exposure to *P. aeruginosa*, *sod-1* (*tm776*), *sod-5* (*tm1146*), and *sod-3* (*tm760*) single mutants elicit heightened avoidance responses to a lawn of *P. aeruginosa*. (C) *sod-1*(*tm776*); *sod-5*(*tm1146*) double mutant does not elicit enhanced pathogen avoidance response compared to *sod-1* (*tm776*) single mutant alone. The behavioral response of *sod-1* (*tm776*); *sod-5* (*tm1146*) is rescued by a 2.9 kb *sod-1* genomic DNA fragment (*sod-1*g). Error bar represents s.e.m. * represents *p* < 0.05. ** represents *p* < 0.01. **** p represents< 0.0001. n.s.: not significant. Determined by one-way ANOVA Tukey's multiple comparison.

We began our study by investigating the role of additional superoxide dismutases in *C. elegans*. Deletion in *sod-1* contributes to a heightened pathogen avoidance response [22]. Therefore, we first obtained deletion mutations of *sod-2*, *sod-3*, *sod-4*, and *sod-5* (Fig. 1A). We then exposed the mutant animals to a lawn of *P. aeruginosa* on a petri dish and compared their *P. aeruginosa* lawn avoidance phenotypes (Fig. 1B). We found that *sod-2* (*gk257*) and *sod-4* (*gk101*) elicited phenotypes similar to wild type. In contrast, we found that *sod-3* (*tm760*) and *sod-5* (*tm1146*) showed enhanced pathogen avoidance responses at 7 h. However, the *P. aeruginosa* avoidance responses of *sod-3* (*tm760*) and *sod-5* (*tm1146*) were not as strong as that of *sod-1* (*tm776*) (Fig. 1B). Since both zinc–copper superoxide dismutase SOD-1 and SOD-5 play roles in regulating *C. elegans* avoidance response to *P. aeruginosa*, we generated a *sod-1* and *sod-5* double mutant and fed the double mutant *P. aeruginosa*. We found that *sod-1* (*tm776*); *sod-5* (*tm1146*) elicited a pathogen avoidance response similar to that elicited by *sod-1* (*tm776*) alone (Fig. 1C). We then introduced a 2.9 kb *sod-1* genomic fragment that encompasses the coding and the 5′ and 3′ regulatory regions of *sod-1* [22] into the *sod-1* (*tm776*); *sod-5* (*tm1146*) double mutant. We were able to rescue the pathogen avoidance response of the *sod-1* (*tm776*); *sod-5* (*tm1146*) double mutant to the wild type level (Fig. 1C). Because a genomic fragment of *sod-1* rescues the pathogen avoidance response of *sod-1*; *sod-5*, SOD-5 may act as an auxiliary isoform to regulate *C. elegans* pathogen response.

**Supp. Fig. 1:**
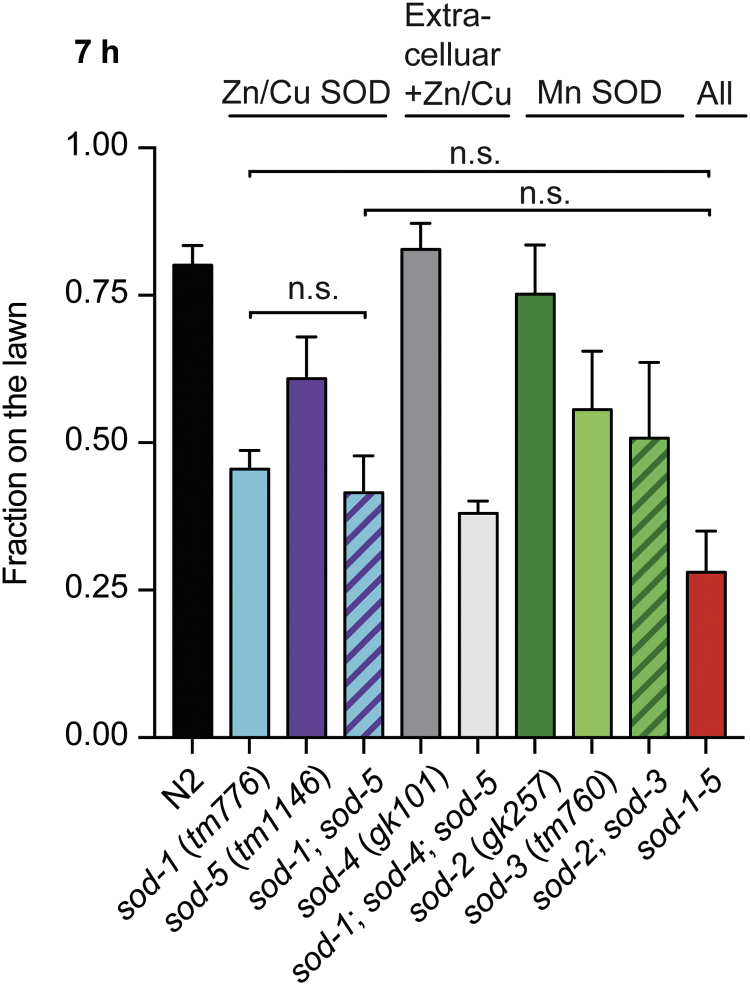
*P. aeruginosa* avoidance response of all superoxide dismutase mutants at 7 h. *N* > 30 for each experiment. n.s.: not significant, as determined by one-way ANOVA Tukey's multiple comparison.

Finally, we compared the pathogen avoidance phenotype of a *sod-1*; *sod-2*; *sod-3*; *sod-4*; *sod-5* quintuple mutant to that of the *sod-1*; *sod-5* double mutant and to the *sod-1* single mutant. We found there are no statistically significant differences between the quintuple mutant, the *sod-1*; *sod-5* double mutant, and the *sod-1* single mutant with respect to avoidance responses to *P. aeruginosa* (Supplementary Fig. 1). Since the quintuple mutant does not further enhance *sod-1* single mutant phenotype, this suggests that SOD-1 is the primary superoxide dismutase to regulate the behavioral avoidance response to *P. aeruginosa*.

### 2.2 SOD-1 and SOD-5 are present in overlapping amphid sensory neurons

**Fig. 2:**
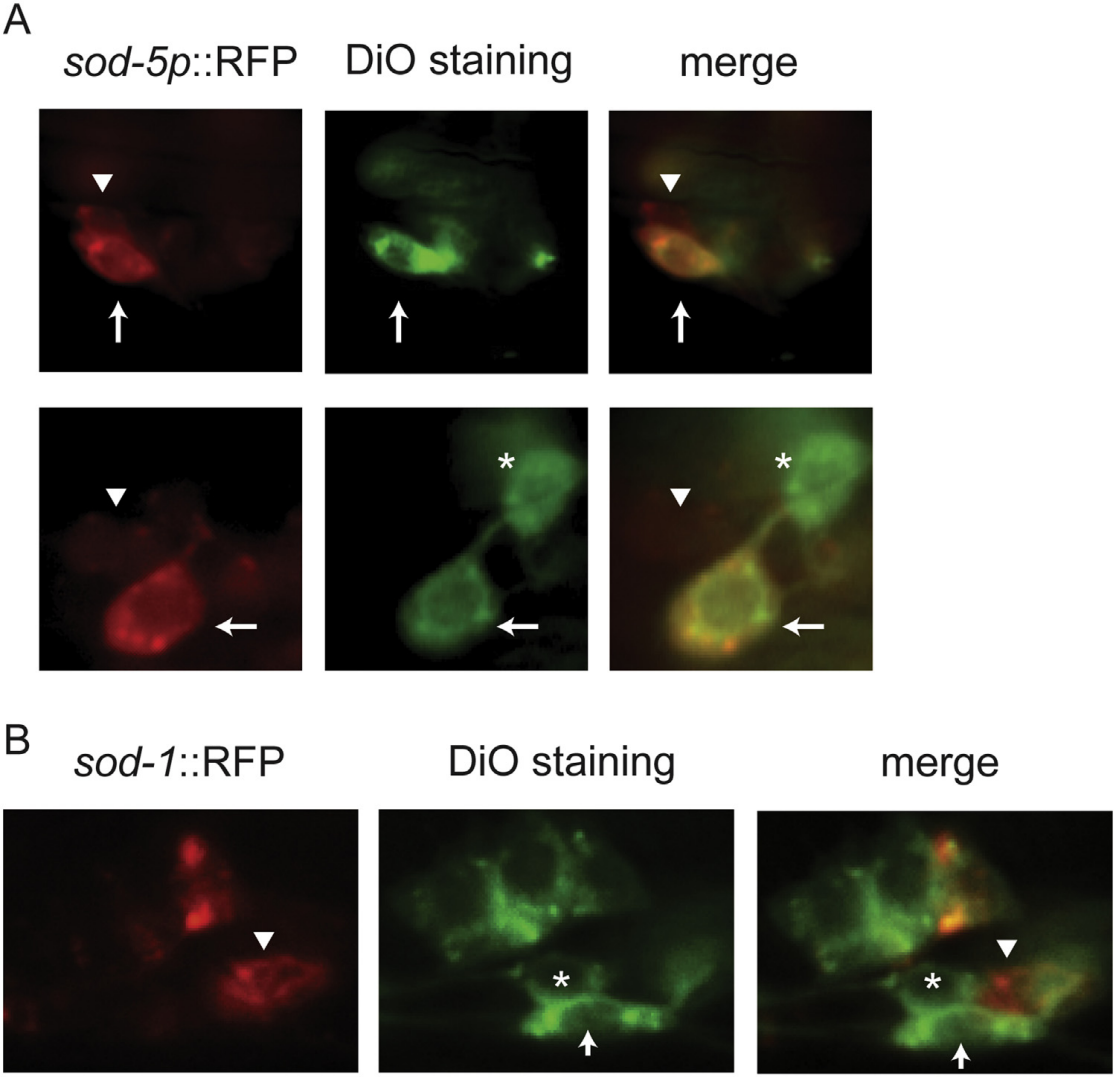
SOD-1 and SOD-5 are present in gustatory neuron ASE. Integrated *sod*-*1p*::*sod*-*1* cDNA::RFP and *sod-5p*::RFP reporters were stained by DiO fluorescence dye. (A) Fluorescence micrographs of *sod-5p*::RFP transgenic animals. *sod-5p*::RFP signals are present in ASE (arrowhead, weak expression) and ASJ (arrow), but not in ASH (star) sensory neurons. Green: DiO staining of amphid sensory neurons. Anterior is to the right. Dorsal is at the top. (B) Fluorescence micrographs of *sod-1p*::*sod-1* cDNA::RFP transgenic animals. SOD-1::RFP signals are present in ASER (arrowhead) and ASJ (arrow), but not in ASH (star) sensory neurons. Green: DiO staining of amphid sensory neurons. Anterior is to the left. Dorsal is at the top.

**Supp. Fig. 2:**
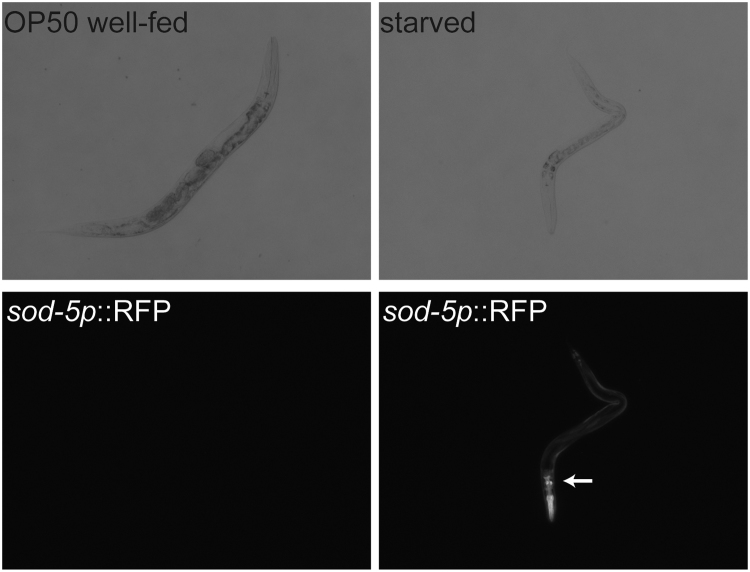
SOD-5 expression is elevated in the nervous system by starvation. Arrow indicates head neurons. Scale bar is 200 µm.

Our data indicate that SOD-5 may act as a redundant zinc–copper superoxide dismutase in the context of pathogen avoidance. If this is the case, SOD-5 is likely present in a similar set of neuronal cells to SOD-1. We therefore sought to investigate the expression pattern of SOD-5. We first generated an integrated reporter strain that contains an RFP reporter driven by the promoter of *sod-5* (*sod-5p*::RFP). We examined the fluorescence signals of *sod-5p*::RFP and detected no obvious *sod-5p*::RFP fluorescence signals when the animals were reared under normal growth condition. However, when the animals were starved, we found *sod-5p*::RFP fluorescence signals in the pharynx and in the nervous system (Supplementary Fig. 2). The fluorescence signals of *sod-5p*::RFP are present in the neuron ganglion posterior to the anterior bulb of the pharynx. Several of the neurons have projections extending anteriorly to the tip of the nose, a morphology that resembles amphid sensory neurons. To determine if SOD-5 is present in the amphid sensory neurons, we performed dye-filing experiments using starved *sod-5p*::RFP animals. We found that *sod-5p*::RFP fluorescence is present in the ASJ neurons. We also observed a weak signal of *sod-5p*::RFP in the ASE neuron pair (Fig. 2A). To further compare the expression pattern of SOD-5 to that of SOD-1, we performed dye-filling experiments using the *sod-1p*::*sod-1* cDNA::RFP reporter strain. Similar to prior results [22], we found that SOD-1 is present in the ASER neuron (Fig. 2B). These results suggest that SOD-1 and SOD-5 are both present in ASER gustatory neurons.

Previous investigation suggests that SOD-5 expression is inducible [42]. Consistent with the prior result, our data show that *sod-5p*::RFP is at undetectable levels under the normal growth condition. We found that the SOD-5 reporter is induced in the nervous system amid starvation and that SOD-5 is present at low levels in the ASE neuron pair. Previous work has also shown that SOD-1 contributes almost 80% of the total *sod* mRNA expression and 80% of the total SOD activities [42]. Together with our results, we reason that SOD-5 likely plays an auxiliary role in mediating the zinc–copper superoxide dismutase–dependent response to *P. aeruginosa*.

### 2.3 SOD-1 is induced by reactive oxygen species in the nervous system and intestine

**Fig. 3:**
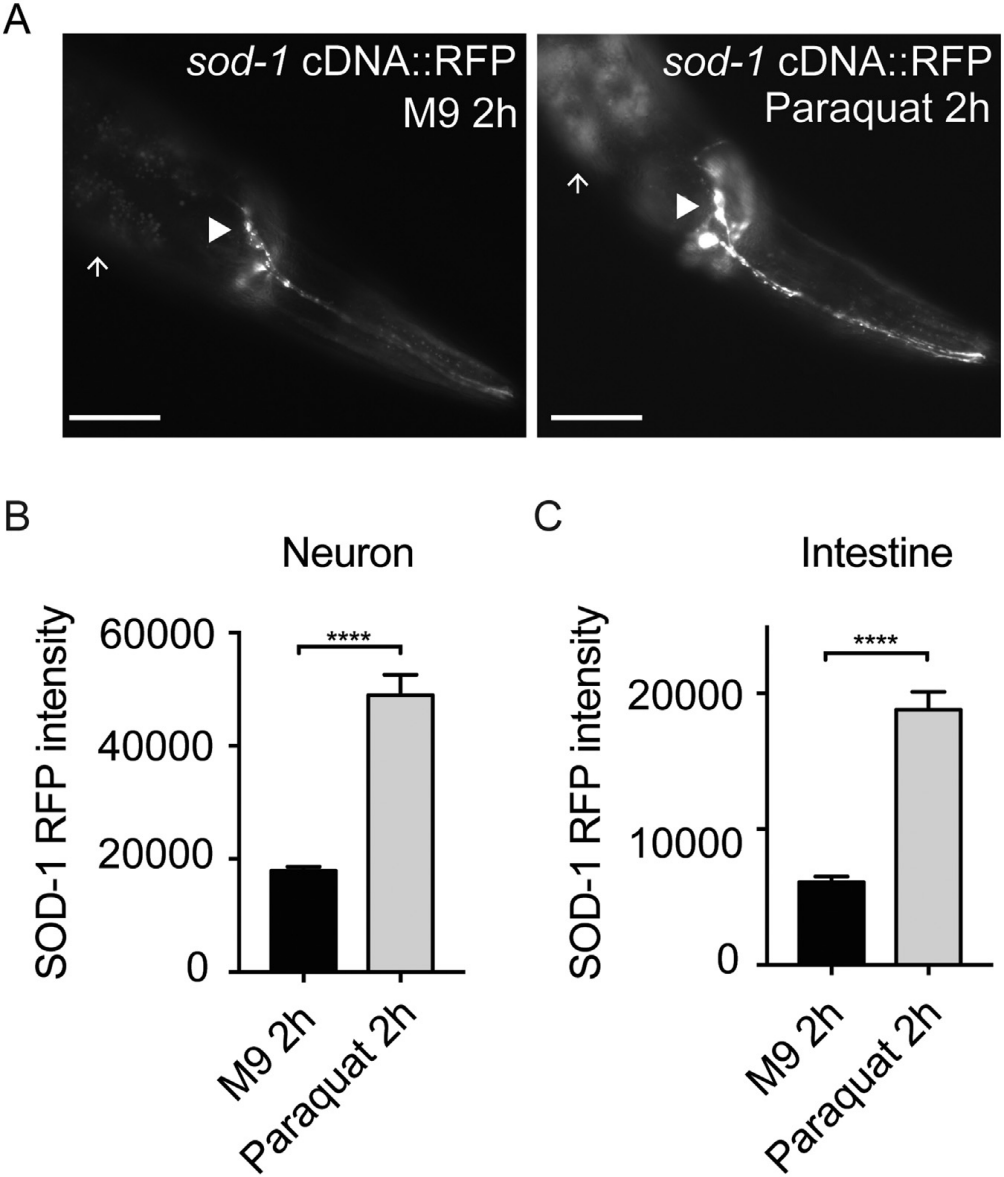
Reactive oxygen species induce SOD-1 expression in the nervous system and intestine. (A) Fluorescence micrographs of integrated *sod*-*1p*::*sod*-*1* cDNA::RFP transgenic animals. SOD-1::RFP level is elevated in the nervous system and intestine by a 2 h treatment with 100 μM paraquat. Arrow: intestine. Arrowhead: ASER neuron. Scale bar represents 50 µm. (B–C) Average fluorescence intensity of SOD-1::RFP in the nervous system (ASER neuron) and intestine. For each experiment in (B) and (C), *N* > 30. **** represents *p* < 0.0001. Determined by one-way ANOVA Tukey's multiple comparison. Error bar: s.e.m.

**Supp. Fig. 3:**
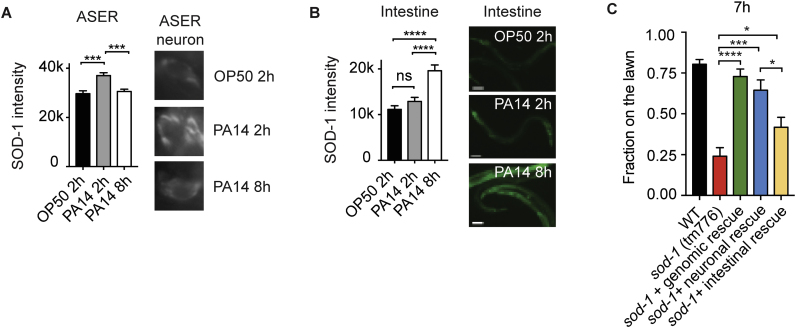
SOD-1 is elevated by *P. aeruginosa* in the nervous system and in the intestine. (A) SOD-1 is elevated by *P. aeruginosa* in the ASER neuron after 2 h. But the expression returns to the baseline at 8 h. (B) SOD-1 is elevated by *P. aeruginosa* in the intestine after 8 h. Scale bar is 50 µm. (C) Pan-neuronal expression of SOD-1 rescues *sod-1* (*tm776*) heightened behavior response to *P. aeruginosa*. Error bar represents s.e.m. * represents *p* < 0.05. *** represents *p* < 0.001. **** represents *p* < 0.0001. n.s.: not significant. Determined by one-way ANOVA Tukey's multiple comparison. *N* > 30 for each experiment.

SOD-1 is an enzyme that converts superoxide into less-toxic hydrogen peroxide and oxygen. We hypothesized that SOD-1 expression is activated by an increase in ROS. To test this, we exposed the SOD-1 fluorescence reporter strain (*sod-1p*::*sod-1* cDNA::RFP) to 100 μM paraquat and measured the fluorescence intensity of SOD-1::RFP. We found that SOD-1 is elevated by paraquat in the nervous system and in the intestine (Fig. 3). We also tested whether exposing *C. elegans* to *P. aeruginosa* induces SOD-1 expression. Similar to previous report [22], we found that the fluorescence signals of SOD-1::GFP are elevated in the ASER neuron after 2 h *P. aeruginosa* exposure. In contrast, intestinal SOD-1::GFP is elevated after 8 h (Supplementary Fig. 3A and B). These results suggest that SOD-1 may act in the nervous system and/or in the intestine in response to an increase in pathogen-induced oxidative stress.

### 2.4 SOD-1 alleviates lethality triggered by pathogen-induced oxidative stress

**Fig. 4:**
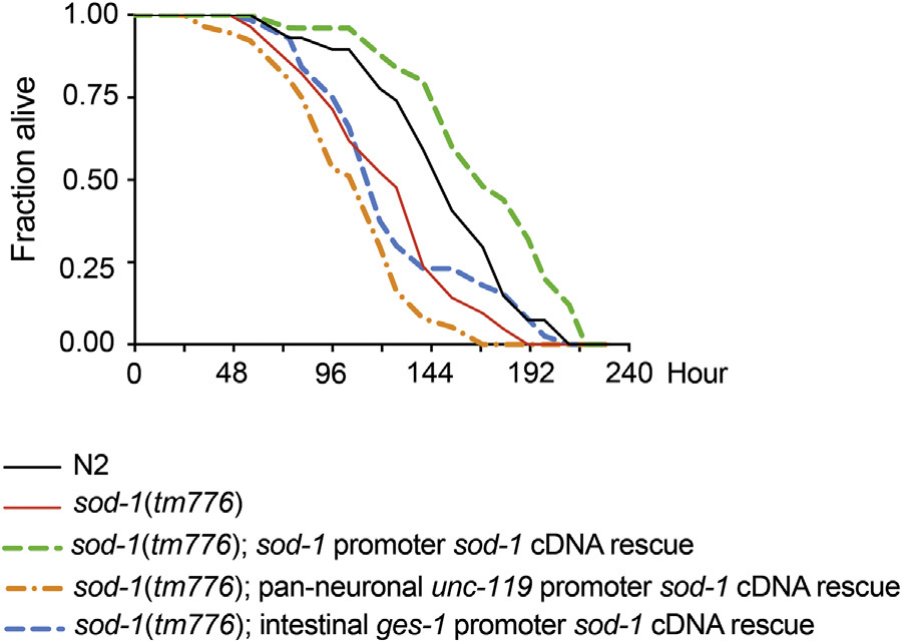
Tissue-specific requirement of SOD-1 in survival after *P. aeruginosa* exposure. After being exposed to *P. aeruginosa* PA14, *sod-1 (tm776*) mutant animals (red) die faster than wild type (black). Endogenous expression of SOD-1 rescues the *sod-1 (tm776)* lethality phenotype and promotes survival (dotted green). Constitutive neuronal expression of SOD-1 (dotted orange) exacerbates the lethality of *sod-1 (tm776)*. Intestinal SOD-1 expression (dotted blue) rescues the *sod-1 (tm776)* lethality phenotype at later time points. For each experiment, *N* > 75.

Exposure to *P. aeruginosa* elevates oxidative stress [22], [29] and contributes to early demise of *C. elegans* [21], [44], [45]. To determine the tissue-specific requirements of SOD-1 in alleviating pathogen-induced oxidative stress, we performed the *P. aeruginosa* survival assay [21]. We introduced wild type, *sod-1* deletion, neuron-specific SOD-1 rescue, intestine-specific SOD-1 rescue, and *sod-1* promoter SOD-1 rescue strains onto a petri dish that contained a lawn of *P. aeruginosa.* We monitored *C. elegans* survival over time (Fig. 4). We found that *sod-1* (*tm776*) died faster than wild type. Expressing SOD-1 under its endogenous promoter (by which SOD-1 is present both in the gut and in the nervous system) in the *sod-1*(*tm776*) mutant restored and extended the survival phenotype. Intestinal SOD-1 rescue extended the survival of *C. elegans* only at later time points. Although pan-neuronal over expression of SOD-1 in the nervous system rescues the heightened avoidance response of *sod-1* mutant (Supplementary Fig. 3C), constitutive pan-neuronal over expression of SOD-1 exacerbated lethality (Fig. 4). This suggests that constitutive SOD-1 elevation in the nervous system is detrimental to *C. elegans*. This also indicates that the levels of neuronal SOD-1 expression need to be carefully controlled.

### 2.5 SOD-1 is activated by guanylyl cyclases GCY-5 and GCY-22 in the ASER neuron

**Fig. 5:**
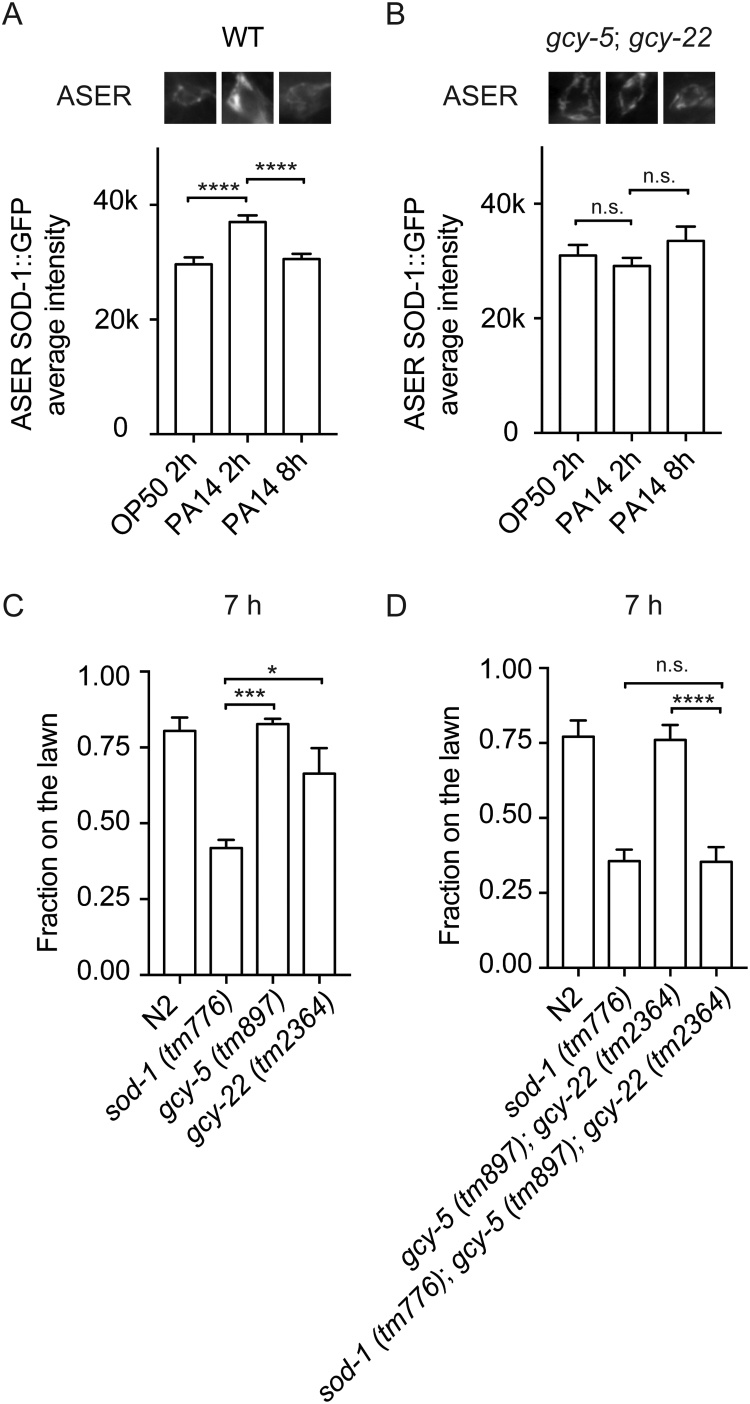
ASER-specific guanylyl cyclases GCY-5 and GCY-22 play a role in SOD-1 induction. (A) SOD-1 expression is elevated in the ASER neuron by *P. aeruginosa* at 2 h. SOD-1 expression returns to the baseline at 8 h. (B) *gcy-5* (*tm857*) and *gcy-22* (*tm2364*) double mutant blocks SOD-1 elevation at 2 h. (C) Pathogen avoidance phenotypes of *gcy-5* (*tm857*) and *gcy-22* (*tm2364*) single mutants. (D) The pathogen avoidance response of *sod-1* (*tm776*); *gcy-5* (*tm857*); *gcy-22* (*tm2364*) triple mutant is similar to that of *sod-1* (*tm776*) single mutant. Error bar represents s.e.m. * represents *p* < 0.05. *** represents *p* < 0.001. **** represents *p* < 0.0001. n.s.: not significant. Determined by one-way ANOVA Tukey's multiple comparison. *N* > 30 for each experiment.

After transferring *C. elegans* to a *P. aeruginosa* diet, SOD-1 expression becomes elevated in the ASER neuron (Fig. 5A). However, the mechanism involved in ASER-specific SOD-1 activation remains unknown. Since the right and left gustatory neurons each express their own set of guanylyl cyclases, we sought to examine whether ASER-specific guanylyl cyclase plays a role in SOD-1 activation. GCY-5 and GCY-22 are guanylyl cyclases uniquely expressed in the ASER neuron [36], [37], [46]. GCY-5 and GCY-22 each contain a single transmembrane domain and a guanylyl cyclase domain. The guanylyl cyclase domain is located near the carboxyl terminus and catalyzes the formation of cyclic GMP. GCY-5 and GCY-22 are proposed to act as gustatory receptors and are responsible for activating the signaling cascade after ligand binding [36], [37]. We introduced a *gcy-5*(*tm897*); *gcy-22*(*tm2364*) double mutant and a *sod-1p*::*sod-1* cDNA::GFP reporter into the same strain. We then switched the strain to a *P. aeruginosa* diet and measured the fluorescence signals of SOD-1::GFP at 2 h and 8 h. We found that the ASER-specific SOD-1::GFP activation is abolished in the *gcy-5*; *gcy-22* double mutant background (Fig. 5B). We then examined whether GCY-5 and GCY-22 regulate *C. elegans* behavioral response to *P. aeruginosa*. We found that neither the single mutants of *gcy-5* and *gcy-22* nor the double mutant of *gcy-5*; *gcy-22* elicited a heightened aversive response to *P. aeruginosa* (Fig. 5C and D). Since the *gcy-5*; *gcy-22* double mutant abolished ASER-specific induction of SOD-1, we reasoned that SOD-1 likely acts downstream of GCY-5 and GCY-22. Therefore, we generated the *sod-1*(*tm776*); *gcy-5*(*tm897*); *gcy-22*(*tm2364*) triple mutant. We compared the pathogen avoidance phenotype of the *sod-1*; *gcy-5*; *gcy-22* triple mutant to that of the *sod-1* single mutant. If SOD-1 acts downstream of GCY-5 and GCY-22, introducing *gcy-5* and *gcy-22* mutations into the *sod-1* background will not change the behavioral response. Indeed, the *sod-1*; *gcy-5*; *gcy-22* triple mutant showed a similar *P. aeruginosa* avoidance response to the *sod-1* single mutant. Our results indicate that GCY-5 and GCY-22 both play a role in activating SOD-1 expression in the ASER neuron when *C. elegans* are exposed to *P. aeruginosa*. However, GCY-5 and GCY-22 do not directly participate in the behavioral response to *P. aeruginosa*.

### 2.6 NPR-1 promotes SOD-1 attenuation in the nervous system

**Fig. 6:**
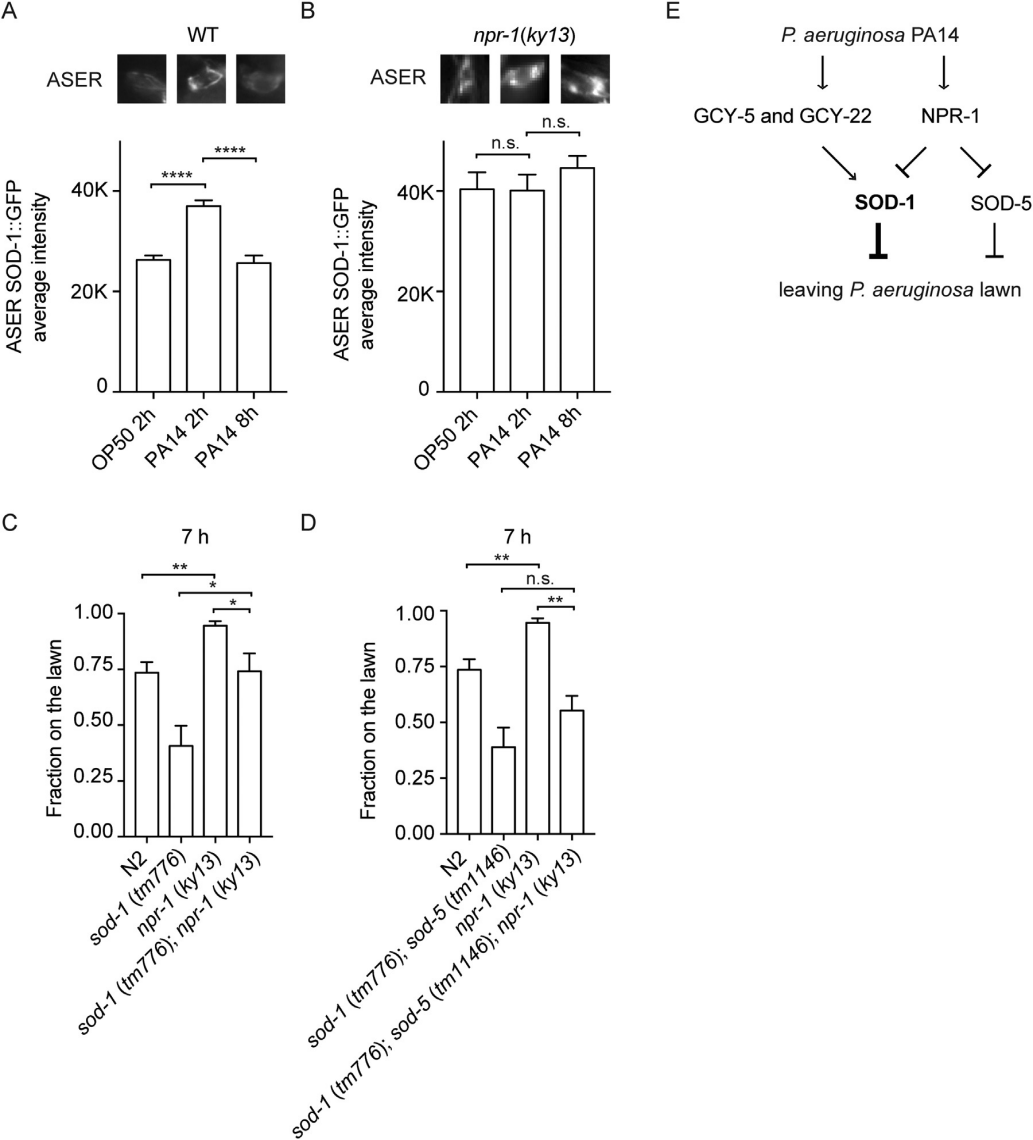
Neuropeptide receptor NPR-1 modulates superoxide dismutase levels and superoxide dismutase–dependent pathogen avoidance response. (A) SOD-1 is elevated in the gustatory neuron ASER by *P. aeruginosa* at 2 h. SOD-1 expression returns to the baseline at 8 h. (B) The temporal regulation of SOD-1 is abolished by the lack of NPR-1. In *npr-1 (ky13*) null mutant, SOD-1 level remains constantly elevated in the ASER neuron. (C) *sod-1* (*tm776*); *npr-1* (*ky13*) double mutant elicits intermediate pathogen avoidance response compared to *sod-1* (*tm776*) and *npr-1* (*ky13*) single mutants. (D) *sod-1* (*tm776*); *sod-5* (*tm1146*); *npr-1* (*ky13*) triple mutant elicits a similar pathogen avoidance response to *sod-1* (*tm776*); *sod-5* (*tm1146*) double mutant. (E) Guanylyl cyclases (GCY-5 and GCY-22) and neuropeptide receptor (NPR-1) act antagonistically to regulate Zn/Cu superoxide dismutase expression in the ASER neuron. Error bar represents s.e.m. * represents *p* < 0.05. ** represents *p* < 0.01. **** p represents < 0.0001. n.s.: not significant. Determined by one-way ANOVA Tukey's multiple comparison. *N* > 30 for each experiment.

Neuropeptide receptor NPR-1 is present in the gustatory neuron ASER [40]. We first tested whether NPR-1 regulates SOD-1 expression in the ASER neuron. We introduced the *npr-1* (*ky13*) mutation into the *sod-1p*::*sod-1* cDNA::GFP reporter line, fed the animals *P. aeruginosa*, and measured the SOD-1::GFP signals. In wild type, SOD-1 expression becomes elevated in the ASER neuron. After 8 h, SOD-1 expression returns to the baseline in the ASER neuron (Fig. 6A). In contrast, the temporal regulation of SOD-1 is abolished by the lack of NPR-1. In the *npr-1* (*ky13*) background, SOD-1 remained constantly elevated throughout the time course of our experiments (Fig. 6B). These results suggest that NPR-1 plays a role in promoting SOD-1 reduction.

### 2.7 NPR-1 inhibits SOD-1– and SOD-5–dependent behavioral response to pathogen

SOD-1 deletion and NPR-1 null mutations elicit opposite *P. aeruginosa* avoidance phenotypes [21], [22], [26]. To determine whether NPR-1 and SOD-1 have a genetic epistasis relationship in *C. elegans* behavioral response to *P. aeruginosa*, we generated *npr-1* (*ky13*); *sod-1* (*tm776*) double mutants, fed the double mutants *P. aeruginosa*, and observed the *P. aeruginosa* avoidance behavior. Compared to *sod-1* and *npr-1* single mutants, the *npr-1*; *sod-1* double mutant elicited an intermediate behavioral response at 7 h (Fig. 6C). Our data suggest that SOD-5 acts as an auxiliary isoform of zinc–copper superoxide dismutase in *C. elegans* (Figs. 1C and 2). We reasoned that the intermediate pathogen avoidance response of the *npr-1* and *sod-1* double mutant might be contributed by the remaining superoxide dismutase activity of SOD-5. Therefore, we generated a *sod-1*(*tm776*); *sod-5*(*tm1146*); *npr-1*(*ky13*) triple mutant. We found that the *sod-1*; *sod-5*; *npr-1* triple mutant elicited a behavioral response comparable to that elicited by the *sod-1*; *sod-5* double mutant (Fig. 6D). This suggests that zinc–copper superoxide dismutase isoforms SOD-1 and SOD-5 act downstream of NPR-1 to regulate the behavioral response to pathogen (Fig. 6E).

Together, our results demonstrate that superoxide dismutase mediates *C. elegans* pathogen avoidance behavior. Among all five isoforms of superoxide dismutase in *C. elegans*, SOD-1 plays a primary role in mediating the behavioral response to *P. aeruginosa* (Fig. 1). SOD-1 is elevated in the intestine and in the nervous system by oxidative stress and *P. aeruginosa* (Fig. 3 and Supplementary Fig. 3). Constitutive neuronal expression of SOD-1 exacerbates the lethality after prolonged exposure to *P. aeruginosa* (Fig. 4). SOD-1 expression becomes elevated in the ASER neuron after 2 h exposure to *P. aeruginosa*. After 8 h, SOD-1 expression returns to the baseline (Supplementary Fig. 3 and Fig. 5A). Although ASER-specific guanylyl cyclases GCY-5 and GCY-22 do not directly mediate the behavioral response to pathogen, GCY-5 and GCY-22 activate superoxide dismutase SOD-1 expression in the ASER neuron (Fig. 5). In contrast, neuropeptide receptor NPR-1 reduces SOD-1 expression in the ASER neuron (Fig. 6). The zinc–copper superoxide dismutase isoform SOD-5 plays a role in mediating pathogen avoidance response (Fig. 1). Because SOD-5 is normally expressed at an undetectable level (Fig. 2 and Supplementary Fig. 2), SOD-5 is likely acting as an auxiliary zinc–copper superoxide dismutase.

## 3 Discussion

The data presented here establish a central role for zinc–copper superoxide dismutase in the pathogen-induced oxidative stress response amid gut dysbiosis. When *C. elegans* are switched to the *P. aeruginosa* diet, both SOD-1 and SOD-5 promote *P. aeruginosa* feeding (Fig. 1). SOD-1 and SOD-5 are present in overlapping sensory neurons, including ASER, a gustatory neuron that has been previously shown to play a role in extending *P. aeruginosa* feeding [22] (Fig. 2). We found that *sod-1* mutant animals showed a stronger aversive response to *P. aeruginosa* than *sod-5* mutant animals. The *sod-1; sod-5* double mutant did not show an enhancement of the aversive behavior when compared to the *sod-1* single mutant. Most importantly, we were able to rescue the *sod-1; sod-5* double mutant phenotype using a *sod-1* genomic fragment (Fig. 1). In addition, we found that SOD-5 is normally expressed at low levels (Supplementary Fig. 2). Previous research has found that SOD-1 contributes almost 80% of the total *sod* mRNA expression as well as 80% of the total SOD activity in *C. elegans* [42]. Together, these data suggest that SOD-1 is the primary zinc–copper superoxide dismutase isoform to mediate the behavioral response to pathogen-induced oxidative stress.

Oxidative stress activates SOD-1 expression in the nervous system and in the intestine (Fig. 3). However, our results show that constitutive SOD-1 overexpression in the nervous system has an adverse effect on animal survival (Fig. 4). This is likely because SOD-1 acts in the nervous system to delay the aversive response to *P. aeruginosa* [22]. This suggests that neuronal expression of SOD-1 has to be carefully controlled. Indeed, we found that SOD-1 is initially elevated by *P. aeruginosa* via ASER-specific GCY-5 and GCY-22 guanylyl cyclases (Fig. 5). After extended feeding on *P. aeruginosa*, SOD-1 expression returns to the baseline (Figs. 5A and 6A). The down-regulation of SOD-1 is mediated by neuropeptide receptor NPR-1 (Fig. 6). Based on these results, we propose that superoxide dismutase SOD-1 is activated by *P. aeruginosa* in the gustatory neuron via guanylyl cyclases GCY-5 and GCY-22. The increase in SOD-1 expression allows *C. elegans* to cope with elevated oxidative stress. However, prolonged SOD-1 elevation in the nervous system extends *P. aeruginosa* feeding and is detrimental to *C. elegans*. It is possible that intestine-derived signals, such as neuropeptides, may act through neuropeptide receptor NPR-1 to facilitate the reduction of SOD-1 and to further fine-tune the behavioral response to pathogens (Fig. 6E).

After extended feeding, *P. aeruginosa* is accumulated in the intestine [26]. We reason that pathogen-induced oxidative stress likely originates in the intestine [2], [27], [29]. Unexpectedly, intestinal SOD-1 overexpression only marginally extended the survival of *C. elegans* (Fig. 4). We found that only by expressing SOD-1 in the nervous system and intestine simultaneously (using the *sod-1* promoter) could we restore the SOD-1 dependent phenotype. These results suggest that there is a possible interplay between the gut and the nervous system in the SOD-1–dependent pathogen response. The underlined molecular mechanism remains to be identified in future studies.

Together, our findings demonstrate that zinc–copper superoxide dismutase SOD-1 plays a primary role in mediating the behavioral response amid gut dysbiosis. SOD-1 expression is regulated by guanylyl cyclases GCY-5 and GCY-22 and neuropeptide receptor NPR-1 in the gustatory neuron ASER. The fine-tuning of behavioral plasticity by the gustatory system via the regulation of superoxide dismutase amid microbe-induced oxidative stress demonstrates a novel strategy to benefit survival. Recent investigations have suggested that consumption of diets that are rich in sugar and fat often contributes to gut dysbiosis. In turn, the sugar- and fat-rich diets activate metabolic endotoxemia and oxidative stress and further negatively impact the function of the brain [47], [48]. The superoxide dismutase–dependent molecular machinery offers the initial evidence for how the nervous system responds to oxidative stress amid gut dysbiosis. Our findings may provide the molecular mechanisms that are conserved across different species.

## 4 Materials and methods

### 4.1 Strains

*C. elegans* strains were maintained at 20 °C using standard methods [14]. Strains were maintained at 20 °C, then shifted to 22.5 °C for *P. aeruginosa* lawn avoidance assays. All of the superoxide dismutase deletion mutants, *gcy-5*(*tm897*), and *gcy-22*(*tm2364*) were obtained from the Caenorhabditis Genetics Center and backcrossed six times to N2 prior to analysis. Transgenic strains were isolated by microinjecting plasmids (typically at 50–100 μg/mL), together with one of the following co-injection markers—*myo-2p::*mstrawberry, *unc-122p::*GFP, and *unc-122p::*mcherry—in wild-type or mutant animals. UV integration of the extrachromosomal array was performed following the protocol originated by S. Mitani [49]. The integrated lines were then backcrossed six times to N2 prior to the analysis.

### 4.2 *P. aeruginosa* avoidance assay

A 100 mL solution of LB was inoculated with a single colony of *P. aeruginosa* PA14 and grown overnight without shaking at 37 °C until O.D. reached 0.2–0.3. 30 μL of this culture was used to seed the center of the 100 mm NGM plate. Seeded plates were incubated for 24 h at room temperature (22.5 °C) prior to the experiment. Approximately 30 animals (young adults) were transferred onto plates containing the *P. aeruginosa* PA14 lawn at 22.5 °C, and lawn occupancy was measured at the indicated times. Three plates of each genotype were performed in each experiment, and all experiments were performed at least three times. Upon being transferred to the *P. aeruginosa*–containing plates, animals explored the plate for about 10–15 min until they found the bacterial lawn and then remained in the lawn. Subsequently, lawn occupancy was measured over time as the lawn avoidance behavior was observed [21].

### 4.3 *P. aeruginosa* pathogenesis survival assay

Survival of *C. elegans* on *P. aeruginosa* PA14 was set up as described previously [21] with the following modifications: 10 μL of the *P. aeruginosa* PA14 culture was used to seed 35-mm Slow-Killing Assay plates. Seeded plates were incubated for 24 h at 22.5 °C. Young adult animals were then transferred to the *P. aeruginosa* PA14 plates, which were maintained at 22.5 °C throughout the survival assay. Animals were transferred to fresh plates every two days that were seeded at the beginning of the experiments. Because of potential adverse effects [50], we did not add 5-fluorodeoxyuridine to our culture media. Animals were scored as dead when they no longer responded to the repeated touch of a platinum wire.

### 4.4 SOD-1 reporter strain activation by paraquat

*sod-1p*::*sod-1* cDNA::RFP was cloned as described previously [22]. The plasmid was injected, integrated, and backcrossed six times to N2 prior to the analysis. The *sod-1p*::*sod-1* cDNA::RFP reporter strain was soaked with 100 μM paraquat in 500 μL M9 buffer for 2 h in a 1.5 mL tube shaking on a nutating mixer. The animals were immediately mounted on slides for imaging after three quick rinses using 500 μL M9 buffer.

### 4.5 Molecular cloning

The genomic region of *sod-1* was amplified by PCR using primers 5′- GAACACCAAACCGGACTGACCAAGT − 3′ and 5′- GTTTATGACGCAAAGCGTACGGACAATCTC − 3′. The 2.9 kb genomic fragment was cloned into a Topo® (Invitrogen) vector. The *sod-5* promoter region and part of the first exon of *sod-5* were amplified by using a 5′ primer containing 5′- GGAAACATCTTTCACGCTGCTGCAACAC − 3′ and a 3′ primer containing 5′- CTGAT ATTGCCAATGCCGTTCTT CCA CA − 3′. The 873 bp fragment was subsequently cloned using *Sph*I/*Kpn*I sites into the modified pPD95.75 vector (Fire Lab Vector Kit, Addgene) that contains RFP to generate the *sod-5* translational RFP reporter. *sod-1* cDNA clones were gifts from Y. Kohara (yk524g1, yk593d7, yk1381e03, and yk1715f05). Detailed primer sets and methods used for cloning are available upon request.

### 4.6 Microscopy

Animals were mounted in M9 with levamisole (10 mM) onto slides with a 3% agarose pad. The slides were viewed using an AxioImager Z1 fluorescence microscope (Zeiss) with 10x/0.25, 40x/0.75, and 63x/1.4 (oil) objectives. The fluorescence signals were recorded by a CCD camera in a 16-bit format without saturation. The images were captured and analyzed by ProgRes imaging software. Fluorescence intensity was measured and calculated using Image-Pro software.

### 4.7 Statistical analysis

Statistical analysis was performed using GraphPad Prism software.

## References

[1] Honda K., Littman D.R. (2016). The microbiota in adaptive immune homeostasis and disease. Nature.

[2] Hooper L.V., Littman D.R., Macpherson A.J. (2012). Interactions between the microbiota and the immune system. Science.

[3] Jostins L. (2012). Host-microbe interactions have shaped the genetic architecture of inflammatory bowel disease. Nature.

[4] Ley R.E., Turnbaugh P.J., Klein S., Gordon J.I. (2006). Microbial ecology: human gut microbes associated with obesity. Nature.

[5] Nicholson J.K. (2012). Host-gut microbiota metabolic interactions. Science.

[6] Qin J. (2012). A metagenome-wide association study of gut microbiota in type 2 diabetes. Nature.

[7] Biagi E. (2016). Gut microbiota and extreme longevity. Curr. Biol..

[8] Bravo J.A. (2011). Ingestion of Lactobacillus strain regulates emotional behavior and central GABA receptor expression in a mouse via the vagus nerve. Proc. Natl. Acad. Sci. USA.

[9] Cryan J.F., Dinan T.G. (2012). Mind-altering microorganisms: the impact of the gut microbiota on brain and behaviour. Nat. Rev. Neurosci..

[10] Diaz Heijtz R. (2011). Normal gut microbiota modulates brain development and behavior. Proc. Natl. Acad. Sci. USA.

[11] Buffington S.A. (2016). Microbial reconstitution reverses maternal diet-induced social and synaptic deficits in offspring. Cell.

[12] Hasegawa S. (2015). Intestinal dysbiosis and lowered serum lipopolysaccharide-binding protein in Parkinson's disease. PLoS One.

[13] Sampson T.R. (2016). Gut microbiota regulate motor deficits and neuroinflammation in a model of parkinson's disease. Cell.

[14] Brenner S. (1974). The genetics of Caenorhabditis elegans. Genetics.

[15] Dirksen P. (2016). The native microbiome of the nematode Caenorhabditis elegans: gateway to a new host-microbiome model. BMC Biol..

[16] Samuel B.S., Rowedder H., Braendle C., Felix M.A., Ruvkun G. (2016). Caenorhabditis elegans responses to bacteria from its natural habitats. Proc. Natl. Acad. Sci. USA.

[17] Labrousse A., Chauvet S., Couillault C., Kurz C.L., Ewbank J.J. (2000). Caenorhabditis elegans is a model host for Salmonella typhimurium. Curr. Biol..

[18] Mahajan-Miklos S., Tan M.W., Rahme L.G., Ausubel F.M. (1999). Molecular mechanisms of bacterial virulence elucidated using a Pseudomonas aeruginosa-Caenorhabditis elegans pathogenesis model. Cell.

[19] Irazoqui J.E. (2010). Distinct pathogenesis and host responses during infection of C. elegans by P. aeruginosa and S. aureus. PLoS Pathog..

[20] Couillault C., Ewbank J.J. (2002). Diverse bacteria are pathogens of Caenorhabditis elegans. Infect. Immun..

[21] Chang H.C., Paek J., Kim D.H. (2011). Natural polymorphisms in C. elegans HECW-1 E3 ligase affect pathogen avoidance behaviour. Nature.

[22] Horspool A.M., Chang H.C. (2017). Superoxide dismutase SOD-1 modulates C. elegans pathogen avoidance behavior. Sci. Rep..

[23] Lee K., Mylonakis E. (2017). An intestine-derived neuropeptide controls avoidance behavior in caenorhabditis elegans. Cell Rep..

[24] Cabreiro F. (2013). Metformin retards aging in C. elegans by altering microbial folate and methionine metabolism. Cell.

[25] MacNeil L.T., Watson E., Arda H.E., Zhu L.J., Walhout A.J. (2013). Diet-induced developmental acceleration independent of TOR and insulin in C. elegans. Cell.

[26] Reddy K.C., Andersen E.C., Kruglyak L., Kim D.H. (2009). A polymorphism in npr-1 is a behavioral determinant of pathogen susceptibility in C. elegans. Science.

[27] Chavez V., Mohri-Shiomi A., Garsin D.A. (2009). Ce-Duox1/BLI-3 generates reactive oxygen species as a protective innate immune mechanism in Caenorhabditis elegans. Infect. Immun..

[28] Ha E.M. (2009). Coordination of multiple dual oxidase-regulatory pathways in responses to commensal and infectious microbes in drosophila gut. Nat. Immunol..

[29] Hoeven R., McCallum K.C., Cruz M.R., Garsin D.A. (2011). Ce-Duox1/BLI-3 generated reactive oxygen species trigger protective SKN-1 activity via p38 MAPK signaling during infection in C. elegans. PLoS Pathog..

[30] Muller F.L. (2006). Absence of CuZn superoxide dismutase leads to elevated oxidative stress and acceleration of age-dependent skeletal muscle atrophy. Free Radic. Biol. Med.

[31] Oeda T. (2001). Oxidative stress causes abnormal accumulation of familial amyotrophic lateral sclerosis-related mutant SOD1 in transgenic Caenorhabditis elegans. Hum. Mol. Genet.

[32] Valentine J.S., Doucette P.A., Zittin Potter S. (2005). Copper-zinc superoxide dismutase and amyotrophic lateral sclerosis. Annu Rev. Biochem.

[33] Bargmann C.I., Horvitz H.R. (1991). Chemosensory neurons with overlapping functions direct chemotaxis to multiple chemicals in C. elegans. Neuron.

[34] Chang S., Johnston R.J., Frokjaer-Jensen C., Lockery S., Hobert O. (2004). MicroRNAs act sequentially and asymmetrically to control chemosensory laterality in the nematode. Nature.

[35] Hobert O. (2014). Development of left/right asymmetry in the Caenorhabditis elegans nervous system: from zygote to postmitotic neuron. Genesis.

[36] Ortiz C.O. (2006). Searching for neuronal left/right asymmetry: genomewide analysis of nematode receptor-type guanylyl cyclases. Genetics.

[37] Ortiz C.O. (2009). Lateralized gustatory behavior of C. elegans is controlled by specific receptor-type guanylyl cyclases. Curr. Biol..

[38] de Bono M., Bargmann C.I. (1998). Natural variation in a neuropeptide Y receptor homolog modifies social behavior and food response in C. elegans. Cell.

[39] Gruner M. (2014). Feeding state, insulin and NPR-1 modulate chemoreceptor gene expression via integration of sensory and circuit inputs. PLoS Genet.

[40] Macosko E.Z. (2009). A hub-and-spoke circuit drives pheromone attraction and social behaviour in C. elegans. Nature.

[41] Wang Y., Branicky R., Noe A., Hekimi S. (2018). Superoxide dismutases: dual roles in controlling ROS damage and regulating ROS signaling. J. Cell Biol..

[42] Doonan R. (2008). Against the oxidative damage theory of aging: superoxide dismutases protect against oxidative stress but have little or no effect on life span in Caenorhabditis elegans. Genes Dev..

[43] Yanase S., Onodera A., Tedesco P., Johnson T.E., Ishii N. (2009). SOD-1 deletions in Caenorhabditis elegans alter the localization of intracellular reactive oxygen species and show molecular compensation. J. Gerontol. A Biol. Sci. Med Sci..

[44] Tan M.W., Mahajan-Miklos S., Ausubel F.M. (1999). Killing of Caenorhabditis elegans by Pseudomonas aeruginosa used to model mammalian bacterial pathogenesis. Proc. Natl. Acad. Sci. USA.

[45] Tan M.W., Rahme L.G., Sternberg J.A., Tompkins R.G., Ausubel F.M. (1999). Pseudomonas aeruginosa killing of Caenorhabditis elegans used to identify P. aeruginosa virulence factors. Proc. Natl. Acad. Sci. USA.

[46] Smith H.K. (2013). Defining specificity determinants of cGMP mediated gustatory sensory transduction in Caenorhabditis elegans. Genetics.

[47] Cani P.D. (2008). Changes in gut microbiota control metabolic endotoxemia-induced inflammation in high-fat diet-induced obesity and diabetes in mice. Diabetes.

[48] Noble E.E., Hsu T.M., Kanoski S.E. (2017). Gut to brain dysbiosis: mechanisms linking western diet consumption, the microbiome, and cognitive impairment. Front Behav. Neurosci..

[49] Kage-Nakadai E., Imae R., Yoshina S., Mitani S. (2014). Methods for single/low-copy integration by ultraviolet and trimethylpsoralen treatment in Caenorhabditis elegans. Methods.

[50] Van Raamsdonk J.M., Hekimi S. (2011). FUdR causes a twofold increase in the lifespan of the mitochondrial mutant gas-1. Mech. Ageing Dev..

